# Psychological Benefits of Walking through Forest Areas

**DOI:** 10.3390/ijerph15122804

**Published:** 2018-12-10

**Authors:** Chorong Song, Harumi Ikei, Bum-Jin Park, Juyoung Lee, Takahide Kagawa, Yoshifumi Miyazaki

**Affiliations:** 1 Center for Environment, Health and Field Sciences, Chiba University, 6-2-1 Kashiwa-no-ha, Kashiwa, Chiba 277-0882, Japan; crsong1028@chiba-u.jp (C.S.); ikei0224@ffpri.affrc.go.jp (H.I.); 2 Forestry and Forest Products Research Institute, 1 Matsunosato, Tsukuba, Ibaraki 305-8687, Japan; kagawa@ffpri.affrc.go.jp; 3 Department of Environment and Forest Resources, Chungnam National University, 99 Daehak-ro, Yuseong-gu, Daejeon 34134, Korea; bjpark@cnu.ac.kr; 4 Department of Landscape Architecture, Hankyong National University, 327 Jungang-ro, Anseong-si, Gyeonggi-do 17579, Korea; lohawi@gmail.com

**Keywords:** forests, shinrin-yoku, forest therapy, psychological relaxation, profile of mood state, brief walks, individual difference, trait anxiety

## Abstract

This study aimed to clarify the psychological benefits of brief walks through forest areas. In addition, we aimed to examine the associations between psychological responses and trait anxiety levels. Five-hundred-and-eighty-five participants (mean age, 21.7 ± 1.6 years) were instructed to walk predetermined courses through forest (test) and city (control) areas for 15 min. The Profile of Mood State (POMS) questionnaire and State-Trait Anxiety Inventory were used to assess participants’ psychological responses and trait anxiety levels, respectively. The results revealed that walking through forest areas decreased the negative moods of “depression-dejection”, “tension-anxiety”, “anger-hostility”, “fatigue”, and “confusion” and improved the participants’ positive mood of “vigor” compared with walking through city areas. Furthermore, a significant correlation was found between participants’ trait anxiety levels and their changes in the subscale of “depression-dejection” of POMS after walking through forest areas. A more effective reduction in the feeling of “depression-dejection” after walking through forest areas was observed for participants with high trait anxiety levels than for those with normal and low trait anxiety levels. This study showed the psychological benefits of walking through forest areas and identified a significant correlation between psychological responses to walking through forests and trait anxiety levels.

## 1. Introduction

As individuals are exposed to several stressors in daily life, they attempt to adopt effective methods for coping with stress and for relaxing. Nature-based experiences have relaxing effects, and the therapeutic effect of nature has received increasing attention.

The positive effects of nature on physical and mental health have been recently reported [1,2,3,4], and the concept of natural therapy has been developed. Nature therapy is defined as “a set of practices aimed at achieving ‘preventive medical effects’ through exposure to natural stimuli that render a state of physiological relaxation and boost the weakened immune functions to prevent diseases” [2]. Nature therapy seeks to improve immune functions, prevent illnesses, and maintain and promote health through exposures to nature with consequent attainment of a relaxed state [1,2].

Numerous studies have focused on and demonstrated the effects of forests in mitigating stress states and inducing physiological relaxation [5,6,7,8,9,10,11,12]. Spending time in forests improves immune functions [13,14], and these effects last for approximately 1 month [15]. In addition, experiments with elderly individuals and adults who are at a risk for stress- and lifestyle-related diseases such as high blood pressure, diabetes, and depression found that various activities performed in forests have positive effects [16,17,18,19,20,21,22,23,24].

However, these studies were limited to small sample sizes, and individual differences within these effects have been noted. A previous study revealed the individual differences in changes of blood pressure after walking through forests [25]. Although some participants showed a decrease in blood pressure after walking through forests, others showed an increase. People respond differently even to the same stimuli. Examination of these individual differences using their initial values demonstrated that participants with initial high blood pressures showed a decrease in blood pressures after walking in a forest, whereas those with initial low blood pressures showed an increase; it concluded that exposure to forest environments had a physiological adjustment effect close to an appropriate level [25]. Moreover, one study [26] assessed individual differences using type A behavioral patterns [27,28], which are known to be specific behavior patterns often exhibited by patients with a heart disease. A previous experiment [26] that involved viewing both forest and city landscapes for 15 min compared the change in the participant’s pulse rate. The results revealed that although the pulse rate decreased in the forest compared with that in the city in the group with type B behavior pattern, which is opposite to the type A behavior pattern. However, there was no significant change in the pulse rate in the group with type A behavior pattern. These results show that physiological responses can vary depending on various factors such as initial values and behavioral patterns [25,26].

Regarding psychological aspects, the restorative effects of natural environment, including forests, that are associated with psychological stressors or mental fatigue, decreased depressive symptoms, and improved mood states have been reported [9,11,29,30,31,32,33]. Kaplan and Kaplan [29] have reported that mental fatigue experienced by individuals nowadays could be restored by contact with a natural environment, and Ulrich et al. [30] demonstrated that natural scenery relieves psychological stress. Shin et al. [31] assessed the effect of forest environment on an individual’s psychological health and well-being and the contribution of a forest experience to improved emotional and cognitive health. In relation to the more direct impacts of natural environment in humans, Park et al. [32] showed that walking around and viewing forests improved emotional state, such as tension and anxiety, depression and dejection, anger and hostility, vigor, confusion, and fatigue, leading to psychological relaxation. Morita et al. [33] reported that walking and staying in forests decrease feelings of hostility and depression and increase liveliness.

Individual differences also exist with regard to psychological responses, and this phenomenon requires further clarification. We previously examined individual differences in changes of mood states such as “depression-dejection”, “tension-anxiety”, “anger-hostility”, “fatigue”, “confusion”, and “vigor” after walking and viewing forests and found significant correlations between them and participants’ initial values [34]. However, studies regarding individual differences in psychological responses are insufficient.

With a large sample population, this study aimed to clarify the psychological benefits of brief walks through forest areas. In addition, we assessed the associations among changes in the mood state of “depression-dejection” after walking through forest areas and trait anxiety levels because mental health problems such as depression and high anxiety are common in modern societies.

## 2. Materials and Methods

### 2.1. Participants and Experimental Sites

**Figure 1:**
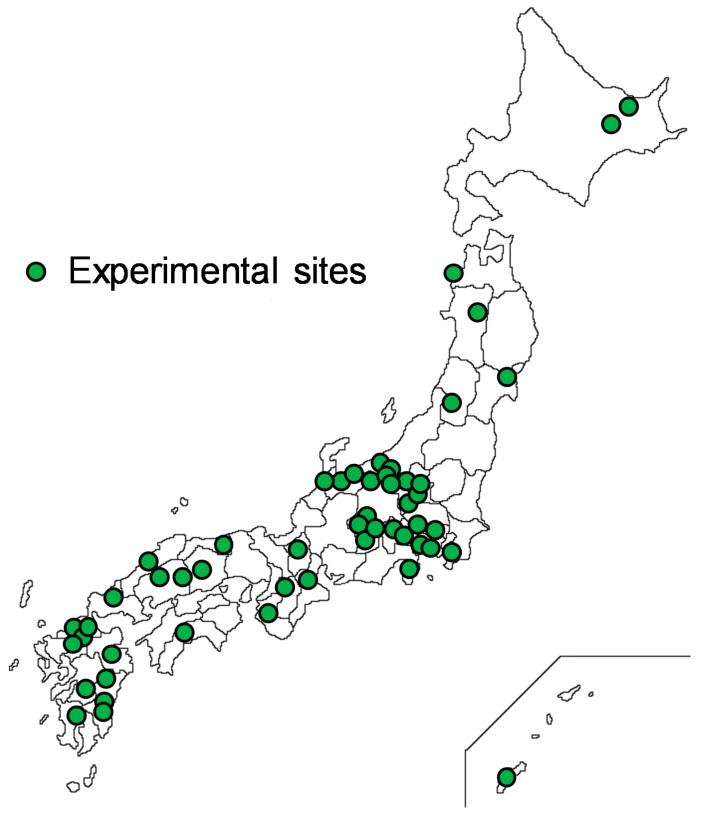
A map showing the distribution of all 52 locations.

From 2005 to 2013, we performed experiments in 52 forest and city areas of Japan. Figure 1 presents a map showing the distribution of all 52 locations. Experiments were conducted in representative forests in each region, and safe, well-maintained forest areas were selected as the experimental sites. City areas were either downtown or near a Japan Railway station. All experiments were conducted during summer between July and September.

Twelve male Japanese university students participated in each experiment (N = 624 participants; 12 participants × 52 areas), and no student reported a history of physical or psychiatric disorder. Of the 624 participants, data from 585 participants (mean age, 21.7 ± 1.6 years) were analyzed. The demographic parameters of the participants are given in Table 1. During the study period, alcohol and tobacco consumption was prohibited, and caffeine consumption was controlled. The study was performed with the approval of the Institutional Ethics Committee of the Forestry and Forest Products Research Institute (project identification code number: 16-558; from 2005 to 2006; 19 areas with 228 participants) and the Ethics Committee of the Center for Environment, Health and Field Sciences, Chiba University (project identification code number: 5; from 2007 to 2013; 33 areas with 396 participants) in Japan.

### 2.2. Experimental Design

**Figure 2:**
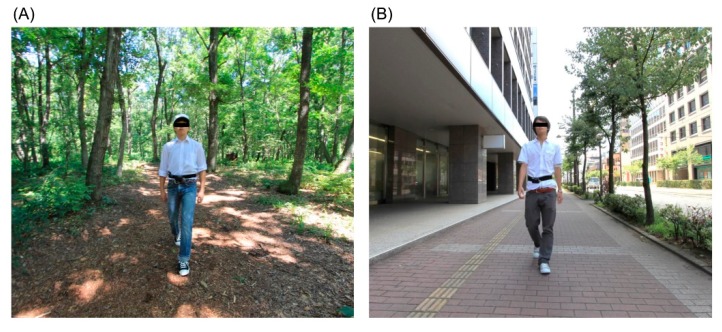
Experimental scenery. (**A**) Forest area and (**B**) city area.

Twelve participants visited the orientation site in each experimental region on the day before (39 areas) or the morning of (13 areas) the experiment. Before initiating the experiments, we explained the aims and procedures of the study to all participants and obtained their written informed consent. Participants were randomly divided into two groups of six persons. On the first day, one group performed the experiment in the forest area, and the other performed the same experiment in the city area. On the second day, participants switched field sites to eliminate order effects. On arrival in the forest or city area, the participants awaited their turn in a waiting room and were eventually taken, one by one, to the experimental site. Each participant took a walk along the given course for approximately 15 min (Figure 2).

### 2.3. Psychological Measurements

For evaluated participants’ mood state, the Profile of Mood State (POMS) questionnaire was used. POMS is a well-established, factor analytically-derived measure of psychological distress, and its high reliability and validity levels have been previously documented [35,36]. POMS simultaneously evaluates six moods: depression and dejection (D), tension and anxiety (T-A), anger and hostility (A-H), fatigue (F), confusion (C), and vigor (V). We used T-scores of POMS for the analysis. In this study, we used the Japanese version of POMS and a short form with 30 questions [37] to reduce the burden on participants. The evaluations of POMS were conducted before and after walking in two areas.

In addition, the State-Trait Anxiety Inventory (STAI) form JYZ was used to assess the participants’ trait anxiety level. STAI is a self-reported tool that measures the presence and severity of current symptoms of anxiety and a generalized propensity to be anxious [38]. Unlike state anxiety, which is a measure of the current state of anxiety that assesses how respondents feel “right now”, trait anxiety is a measure of the relatively stable aspects of “anxiety proneness” as assessed by 20 questions [39]. In our study, scores of ≥44 were considered to be the high trait anxiety group, scores of ≤43 and ≥33 were considered to be the normal trait anxiety group, and scores of ≤32 were considered to be the low trait anxiety group.

### 2.4. Data Analysis

The Wilcoxon signed-rank test was used to compare psychological responses after walking through the forest and city areas.

Pearson’s correlation test was used to analyze the correlation between scores of the POMS subscales after walking through forest areas (the value after walking through a forest area compared to the value after walking through a city area) and those of trait anxiety of STAI.

Mann–Whitney U test was used to between participants with high trait anxiety levels and those with normal and low trait anxiety levels.

Statistical analyses were performed using the Statistical Package for Social Sciences (SPSS version 20.0, SPSS Inc., Chicago, IL, USA). In all cases, *p*-values of <0.05 were considered to be statistically significant.

## 3. Results

**Figure 3:**
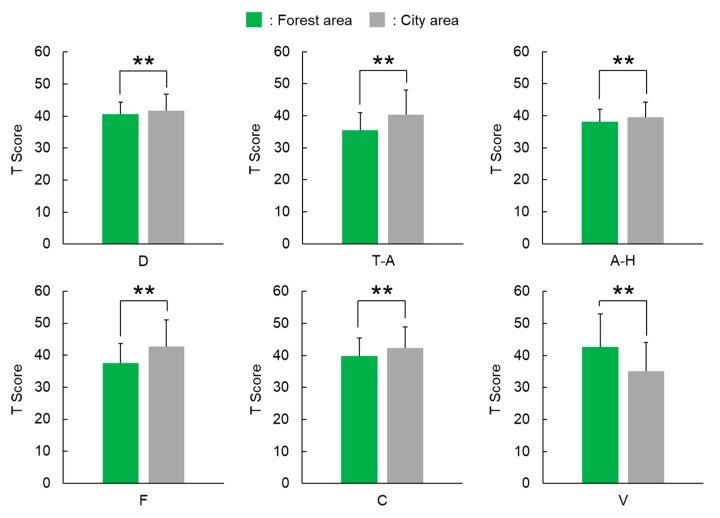
Scores of the Profile of Mood States after walking through forest and city areas. (D), depression-dejection; (T-A), tension–anxiety; (A-H), anger-hostility; (F), fatigue; (C), confusion; and (V), vigor. *N* = 585; mean ± standard deviation; **, *p* < 0.01 by Wilcoxon signed-rank test.

Significant differences between walking through forest and city areas were observed for all subscales of D, T-A, A-H, F, C, and V (Figure 3). The score of the D subscale was 40.6 ± 3.7 (mean ± standard deviation) after walking through forest areas, which was significantly lower than 41.6 ± 5.3 after walking through city areas (*p* < 0.01). Similar results were obtained for T-A (forest, 35.5 ± 5.5; city, 40.4 ± 7.7; *p* < 0.01), A-H (forest, 38.1 ± 3.9; city, 39.5 ± 4.7; *p* < 0.01), F (forest, 37.5 ± 6.2; city, 42.7 ± 8.4; *p* < 0.01), and C (forest, 39.8 ± 5.6; city, 42.2 ± 6.7; *p* < 0.01) subscales, and a decrease in negative mood state was observed after walking through forest areas. In contrast, regarding the positive mood state of V, the score after walking through forest areas was 42.6 ± 10.4, which was significantly higher than 35.1 ± 8.9 after walking through city areas (*p* < 0.01); thus, an increase in positive mood state was observed after walking through forest areas.

**Figure 4:**
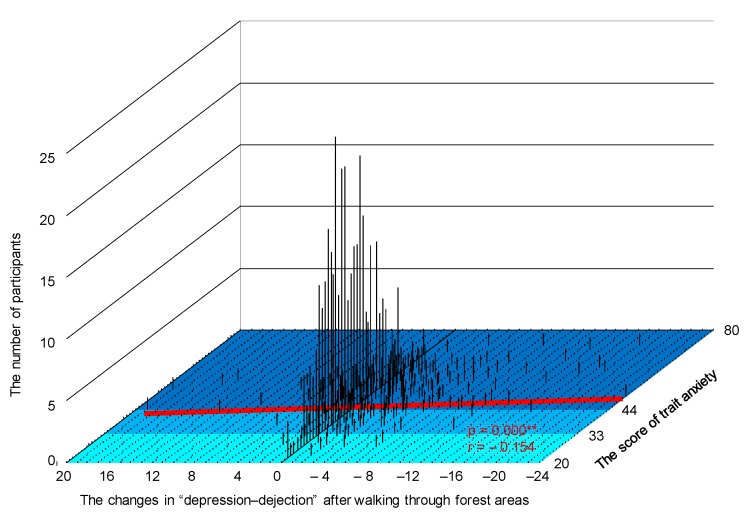
Three-dimensional graph showing the changes in “depression-dejection” after walking through forest areas, trait anxiety scores, and number of participants. *N* = 585, **: *p* < 0.01 by Pearson’s correlation test.

Figure 4 shows a three-dimensional graph in which the *x*-axis denotes the changes after following walking through forest areas, the *y*-axis denotes the trait anxiety scores of STAI, and the *z*-axis denotes the number of participants. A significant correlation was observed between changes in the D subscale after walking through forest areas (the value after walking through a forest area compared to the value after walking through a city area) and the participants’ trait anxiety levels (*p* < 0.01; Figure 4).

Participants with high trait anxiety levels tended to have a more effective reduction in the feeling of “depression-dejection” after walking through forest areas compared with those with normal and low trait anxiety levels (participants with high trait anxiety, *N* = 327; participants with normal and low trait anxiety, *N* = 258; *p* = 0.075).

**Figure 5:**
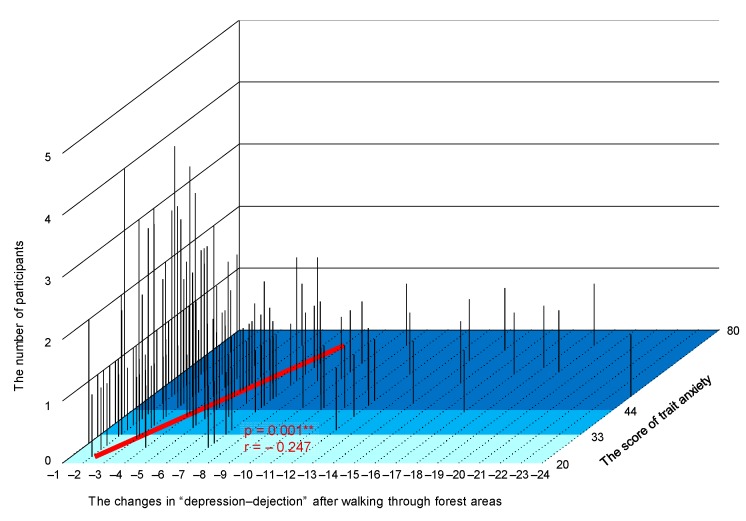
Three-dimensional graph on the changes in “depression-dejection” after walking through forest areas, trait anxiety score, and number of participants in the decreasing group. *N* = 182; **, *p* < 0.01 by Pearson’s correlation test.

Of the 585 participants, 182 participants showed decreased feeling of “depression-dejection” after walking in forests. Meanwhile, 56 participants experienced increased feeling of “depression-dejection,” and 347 participants did not experience any changes. Figure 5 shows the results of participants whose feelings of “depression-dejection” decreased after walking through forest areas. A significant correlation was observed between changes after walking through forest areas and the participants’ trait anxiety levels (*p* < 0.01; Figure 5).

Participants with higher trait anxiety levels tended to show greater decreases than those with normal and low trait anxiety levels (participants with high trait anxiety, *N* = 123; participants with normal and low trait anxiety, *N* = 59; *p* = 0.064).

## 4. Discussion

This study found that walking through forest areas decreased the negative moods of “depression-dejection”, “tension-anxiety”, “anger-hostility”, “fatigue”, and “confusion” and improved the participants’ positive mood of “vigor” compared with walking through city areas. These results, which demonstrate the psychological benefits of forests, are partly consistent with previous findings of the effect of viewing forest scenery or walking in forests [9,11,32]. Park et al. [32] has demonstrated that walking around and viewing forests improved negative emotions, such as depression-dejection, tension-anxiety, anger-hostility, fatigue, and confusion, as well as a positive emotion of vigor, in 168 participants at 14 locations; these results are consistent with our findings. Present study is the first study to use a sample size as large as 585 participants, and the psychological benefits of walking through forests were evident with this larger sample.

A significant correlation was found between participants’ trait anxiety levels and their changes in the “depression-dejection” subscale of POMS after walking through forest areas. Our data revealed that psychological responses can differ depending on a participant’s trait anxiety levels and that those participants with high trait anxiety levels tended to have a more effective reduction in the feeling of “depression-dejection” after walking through forest areas than participants with normal and low trait anxiety levels. Only the feeling of “depression-dejection” had a significant correlation, and no significant correlation was found between the other subscales of POMS. In future studies, this point must be considered. Very few studies have assessed individual differences in psychological responses, and therefore, more researches in this area are required.

More than half of the global population currently lives in urban environments, and 69% of individuals are expected to live in urban areas by 2050 [40,41]. Although urbanization has led to improvements in many areas such as housing, employment, education, equality, quality of living environment, social support, and health services [42], changes that have occurred over a very short period have been very drastic from an evolutionary perspective. Recent research showed that city dwellers are constantly exposed to stressors and that urban living is associated with increased risk of health problems [43,44,45,46]. In particular, mental health problems were profound. Current city dwellers have a 39% higher risk for mood disorders and 21% higher risk for anxiety disorders [44] and higher rates of psychotropic medication prescriptions for anxiety, depression, and psychosis [46]. Therefore, the psychological benefits of walking through forests are very significant, and forest environments are expected to have very important roles in promoting mental health in the future. It is necessary to consider the health policy using nature including forests. Furthermore, urban planners should pay more attention to maintaining and increasing accessible greenery in urban areas. The beneficial effects of nature suggest a simple, accessible, and cost-effective method to improve the quality of life and health of urban residents.

This study had several limitations. Firstly, this study was conducted in representative forests in each region to validate the psychological effect of walking in forest areas. Because the experiments were conducted at 52 different sites, the difference according to region might have affected the result. The effects according to the various characteristics of the forests must be examined in the future. Secondly, to generalize the findings, further studies that include various other demographic groups such as females and individuals with different ages are required. Thirdly, for an overall discussion, verifying the effect of forests using other psychological measurements is necessary to demonstrate the psychological effect of forests. Finally, participants’ prior expectations and experience with forests may influence the results. These limitations should be considered in future research.

## 5. Conclusions

This study demonstrated the psychological benefits of walking through forest areas and revealed a significant correlation between psychological responses and trait anxiety levels.

## Figures and Tables

**Table 1:** Participant demographics.

Parameter	Mean ± Standard Deviation
Total sample number	585
Age (years)	21.7 ± 1.6
Height (cm)	172.4 ± 5.6
Weight (kg)	64.6 ± 9.4
BMI ^1^ (kg/m^2^)	21.7 ± 2.9

^1^ BMI, body mass index.
