# Correction: Song, C.; Ikei, H.; Park, B.J.; Lee, J.; Kagawa, T.; Miyazaki, Y. Psychological Benefits of Walking through Forest Areas. *Int. J. Environ. Res. Public Health* 2018, 15, 2804

**DOI:** 10.3390/ijerph17041316

**Published:** 2020-02-18

**Authors:** Chorong Song, Harumi Ikei, Bum-Jin Park, Juyoung Lee, Takahide Kagawa, Yoshifumi Miyazaki

**Affiliations:** 1 Center for Environment, Health and Field Sciences, Chiba University, 6-2-1 Kashiwa-no-ha, Kashiwa, Chiba 277-0882, Japan; crsong1028@chiba-u.jp (C.S.); ikei0224@ffpri.affrc.go.jp (H.I.); 2 Forestry and Forest Products Research Institute, 1 Matsunosato, Tsukuba, Ibaraki 305-8687, Japan; kagawa@ffpri.affrc.go.jp; 3 Department of Environment and Forest Resources, Chungnam National University, 99 Daehak-ro, Yuseong-gu, Daejeon 34134, Korea; bjpark@cnu.ac.kr; 4 Department of Landscape Architecture, Hankyong National University, 327 Jungang-ro, Anseong-si, Gyeonggi-do 17579, Korea; lohawi@gmail.com

The authors wish to add the following corrections to their paper published in the *International Journal of Environmental Research and Public Health* [1]. When calculating the *T*-score of the Profile of Mood States (POMS) data, an error occurred. The following change should be made to the Figure 3, Figure 4 and Figure 5 and their explanation in the published article. The change does not affect the conclusions of the article in any way. 

Figure 3 should be replaced with the following figure:

Lines 5–14 on page 5 should be replaced with the following text:

Significant differences between walking through forest and city areas were observed for all subscales of D, T-A, A-H, F, C, and V (Figure 3). The score of the D subscale was 40.6 ± 4.0 (mean ± standard deviation) after walking through forest areas, which was significantly lower than 41.7 ± 5.4 after walking through city areas (*p* < 0.01). Similar results were obtained for T-A (forest, 36.1 ± 5.4; city, 41.3 ± 7.7; *p* < 0.01), A-H (forest, 38.1 ± 4.2; city, 39.8 ± 5.2; *p* < 0.01), F (forest, 37.1 ± 6.5; city, 42.9 ± 8.9; *p* < 0.01), and C (forest, 41.6 ± 5.3; city, 44.3 ± 6.9; *p* < 0.01) subscales, and a decrease in negative mood state was observed after walking through forest areas. In contrast, regarding the positive mood state of V, the score after walking through forest areas was 43.8 ± 10.5, which was significantly higher than 35.6 ± 8.7, reported after walking through city areas (*p* < 0.01); thus, an increase in positive mood state was observed after walking through forest areas.

Figure 4 should be replaced with the following figure:

Lines 24–27 on page 5 should be replaced with the following text:

Participants with high-trait anxiety levels have a more effective reduction in the feeling of “depression–dejection” after walking through forest areas compared with those with normal and low-trait anxiety levels (participants with high-trait anxiety, N = 325; participants with normal and low-trait anxiety, N = 260; *p* < 0.05).

Lines 12–20 on page 7 should be replaced with the following text:

A significant correlation was found between participants’ trait anxiety levels and their changes in the “depression–dejection” subscale of POMS after walking through forest areas. Our data revealed that psychological responses can differ depending on a participant’s trait anxiety levels and that those participants with high-trait anxiety levels have a more effective reduction in the feeling of “depression–dejection” after walking through forest areas than participants with normal and low trait anxiety levels. Only the feeling of “depression–dejection” had a significant correlation, and no significant correlation was found between the other subscales of POMS. In future studies, this point must be considered. Very few studies have assessed individual differences in psychological responses and, therefore, more research in this area is required.

Figure 5 should be replaced with the following figure:

Lines 5–13 on page 6 should be replaced with the following text:

Of the 585 participants, 163 participants showed decreased feelings of “depression–dejection” after walking in forests. Meanwhile, 49 participants experienced increased feelings of “depression–dejection,” and 373 participants did not experience any changes. Figure 5 shows the results of participants whose feelings of “depression–dejection” decreased after walking through forest areas. A significant correlation was observed between changes after walking through forest areas and the participants’ trait anxiety levels (*p* < 0.05; Figure 5).

However, there was no significant difference between participants with high-trait anxiety levels and those with normal and low-trait anxiety levels (participants with high-trait anxiety, *N* = 113; participants with normal and low-trait anxiety, *N* = 50; *p* > 0.05).

We apologize for any inconvenience caused to the readers by this error.

## Figures and Tables

**Figure 3:**
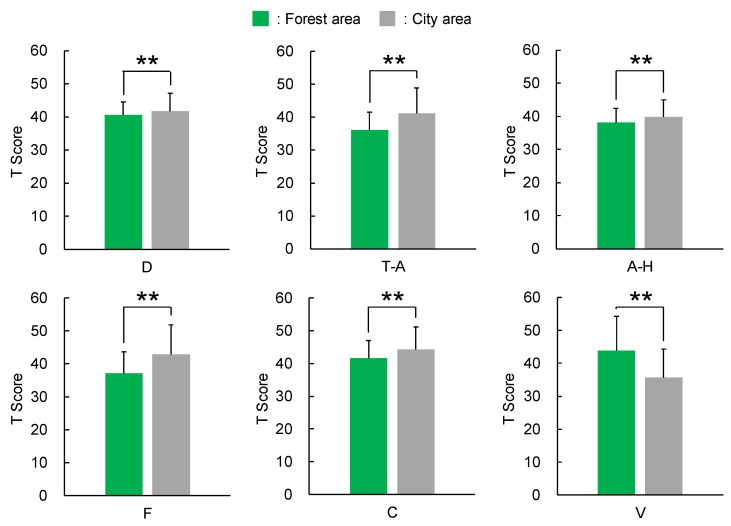
Scores of the Profile of Mood States after walking through forest and city areas. (D), depression–dejection; (T-A), tension–anxiety; (A-H), anger–hostility; (F), fatigue; (C), confusion; and (V), vigor. *N* = 585; mean ± standard deviation; **, *p* < 0.01 by Wilcoxon signed-rank test.

**Figure 4:**
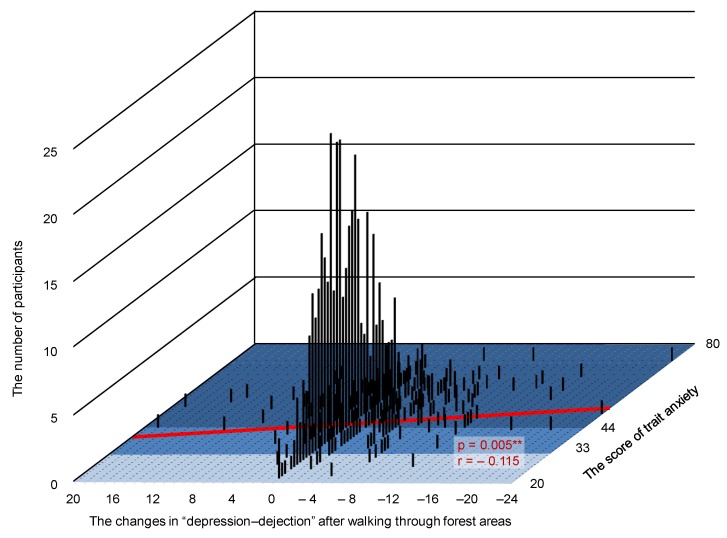
Three-dimensional graph showing the changes in “depression–dejection” after walking through forest areas, trait anxiety scores, and number of participants. *N* = 585, **: *p* < 0.01 by Pearson’s correlation test.

**Figure 5:**
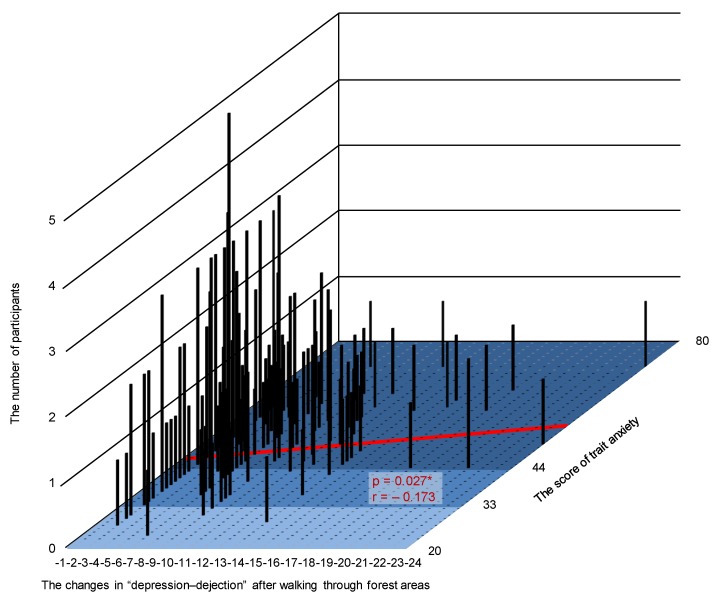
Three-dimensional graph on the changes in “depression–dejection” after walking through forest areas, trait anxiety score, and number of participants in the decreasing group. *N* = 163; *, *p* < 0.05 by Pearson’s correlation test.
